# The TLR4 Agonist Fibronectin Extra Domain A is Cryptic, Exposed by Elastase-2; use in a fibrin matrix cancer vaccine

**DOI:** 10.1038/srep08569

**Published:** 2015-02-24

**Authors:** Ziad Julier, Mikaël M. Martino, Alexandre de Titta, Laura Jeanbart, Jeffrey A. Hubbell

**Affiliations:** 1 Institute of Bioengineering, Ecole Polytechnique Fédérale de Lausanne, 1015, Lausanne, Switzerland; 2 World Premier International Immunology Frontier Research Center, Osaka University, Suita, Osaka 565-0871, Japan; 3 Institute for Chemical Sciences and Engineering, Ecole Polytechnique Fédérale de Lausanne, 1015, Lausanne, Switzerland; 4 Institute for Molecular Engineering, University of Chicago, Chicago, IL 60637, USA; 5 Materials Science Division, Argonne National Laboratory, Argonne, IL 60439, USA

## Abstract

Fibronectin (FN) is an extracellular matrix (ECM) protein including numerous fibronectin type III (FNIII) repeats with different functions. The alternatively spliced FN variant containing the extra domain A (FNIII EDA), located between FNIII 11 and FNIII 12, is expressed in sites of injury, chronic inflammation, and solid tumors. Although its function is not well understood, FNIII EDA is known to agonize Toll-like receptor 4 (TLR4). Here, by producing various FN fragments containing FNIII EDA, we found that FNIII EDA's immunological activity depends upon its local intramolecular context within the FN chain. N-terminal extension of the isolated FNIII EDA with its neighboring FNIII repeats (FNIII 9-10-11) enhanced its activity in agonizing TLR4, while C-terminal extension with the native FNIII 12-13-14 heparin-binding domain abrogated it. In addition, we reveal that an elastase 2 cleavage site is present between FNIII EDA and FNIII 12. Activity of the C-terminally extended FNIII EDA could be restored after cleavage of the FNIII 12-13-14 domain by elastase 2. FN being naturally bound to the ECM, we immobilized FNIII EDA-containing FN fragments within a fibrin matrix model along with antigenic peptides. Such matrices were shown to stimulate cytotoxic CD8^+^ T cell responses in two murine cancer models.

Fibronectin (FN) is a ubiquitous multidomain extracellular matrix (ECM) component and is critically important in numerous ECM-dependent processes such as cell adhesion, migration, growth, and differentiation^1^. Notably, certain functions of FN depend on the presence of the alternatively spliced type III repeats extra domains A (FNIII EDA) and B (FNIII EDB). These extra domains are expressed in the vicinity of developing vessels during embryogenesis^2^,^3^ and later in particular cases such as after tissue injury^4^,^5^,^6^, within tumors^7^,^8^,^9^, and at sites of chronic inflammation such as psoriatic lesions^10^,^11^,^12^. Although expression of FNIII EDA and FNIII EDB is crucial for embryonic development^13^, their postnatal functions remain incompletely elucidated.

One of the most interesting functions of FNIII EDA is its ability to act as an endogenous ligand for the pattern recognition receptor Toll-like receptor 4 (TLR4)^14^ and activate its signaling pathway in a MyD88-dependent manner, which leads to the activation of NF-κB. Importantly, this TLR4-agonizing activity has been shown to contribute to disease progression, specifically in driving fibrosis in scleroderma^15^ and stimulating the inflammatory cascade in psoriatic lesions^10^.

Here, we sought to explore the importance of the local molecular context of FNIII EDA in its immunological activities, as the domain resides in the vicinity of other active FNIII repeats in the natural sequence FNIII 9-10-11-EDA-12-13-14 (Fig. 1a). FNIII 10 contains the well-studied RGD sequence that binds integrins such as α_v_β_3_^16^ and FNIII 9 contains the synergy site PHSRN that is critical for α_5_β_1_ integrin activation^17^. FNIII 11 has been shown to bind anastellin^18^, a small FN type I fragment, increasing its thermolytic digestion. The FNIII 12-13-14 domain is one of the major heparin-binding sites of FN^19^ and has been shown to promiscuously bind growth factors^20^,^21^. In some of FN's biological activities, this molecular context has been shown to be important; for example, the proximal location of the integrin-binding domain and the growth factor binding domain has been shown to lead to synergistic signaling between the ligated integrin and the ligated growth factor receptor, potentiating growth factor signaling^21^,^22^,^23^. Based on these recent findings, we were motivated to investigate the potential importance of the molecular context of FNIII EDA's placement within the FN chain upon its activity in inducing immune responses.

Moreover, FNIII EDA is known to induce the expression of inflammatory cytokines and matrix metalloproteinases (MMPs)^24^, and these activities have been shown to be dependent on the presence of the neighboring domains FNIII11 and FNIII12^24^. For such exposure to occur *in vivo*, it would require FN to be cleaved by a protease, but only the bacterial protease thermolysin from *Bacillus thermoproteolyticus* has been shown to cleave FN close to the C-terminus of FNIII EDA^25^. Because no mammalian protease had been shown to carry out the same function, we were thus also motivated to search for mammalian proteases that might modulate the activity of FNIII EDA by inducing such cleavage.

Finally, because FNIII EDA can induce cellular immune responses including activation of CD8^+^ T cell responses^26^,^27^,^28^,^29^,^30^,^31^, the domain has also been explored as a cancer vaccine adjuvant in mouse models^28^,^30^,^31^. However, FNIII EDA showed potency mostly in combination with other TLR agonists^28^,^31^. Here, because FNIII EDA is naturally displayed bound to the ECM, we hypothesized that its relatively weak adjuvancy could be related to the fact that the domain was delivered as a soluble protein. Therefore, we also explored whether FNIII EDA variants could have better adjuvant potency when bound to the ECM.

## Results

### Production of FN fragments

**Figure 1:**
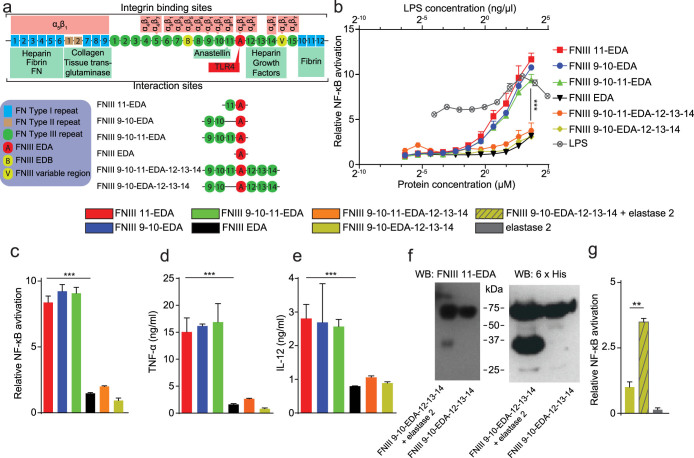
TLR4 activation by FNIII EDA is modulated by the context of its neighboring FNIII domains. (a) FNIII EDA-containing FN fragments produced and of full length FN with some of its interaction sites displayed. (b) Activation of NF-κB in THP1-Blue cells as an indication of TLR agonization in response to various FNIII EDA-containing FN fragments, compared to LPS. The presence of a domain N-terminal to the FNIII EDA domain enhances activation, and the neighboring C-terminal domain abrogates activation. In the absence of the FNIII 12-13-14 domain, activation is comparable to that achieved by LPS. (c) Activation of NF-κB in HEK-Blue TLR4 cells as a cellular bioassay of TLR4 agonization. The presence of a domain N-terminal to the FNIII EDA domain enhances TLR4 agonization, and the neighboring C-terminal domain abrogates TLR4 agonization. In the absence of the FNIII 12-13-14 domain, TLR4 agonization is strong. (d, e) Production of TNF-α (d) or IL-12p70 (e) from murine bone marrow-derived DCs upon stimulation with various FNIII EDA-containing FN fragments. In DCs, the same pattern of activation was observed as with the previous cell lines: the presence of a domain N-terminal to the FNIII EDA domain enhances activation, and the neighboring FNIII 12-13-14 domain abrogates activation. (f) Western blots of the digestion product of FNIII 9-10-EDA-12-13-14 by elastase 2 and undigested control, blotted against the FNIII EDA domain (N-terminal to a predicted elastase 2 cleavage site) and a 6xHis tag (C-terminal). Cleavage was observed to yield two fragments of similar molecular weight, consistent with the presence of elastase 2 cleavage site at position 87 of the 94 amino acid-long FNIII EDA domain. (g) Activation of HEK-Blue TLR4 cells with the digested and undigested FNIII 9-10-EDA-12-13-14. Digestion of FNIII 9-10-EDA-12-13-14 with elastase 2 recovers activation, consistent with elastase 2-dependent cryptic agonization of TLR4. Cellular experiments in c, d, e and f were done in the presence of 10 μg/ml polymixin B, to avoid any influence of potentially contaminating LPS. In c, d, e and f, bars and curves represent mean ± SEM of triplicate cultures from two independent experiments. **P < 0.01; ***P < 0.001.

To investigate the importance of the biomolecular context of FNIII EDA's activity as a TLR4 agonist, we expressed various FN fragments containing FNIII EDA (Fig. 1a). As the simplest construct, FNIII EDA was produced as a single FNIII repeat. Extending N-terminally, FNIII 11 was incorporated to produce FNIII 11-EDA. To further explore N-terminal extensions, FNIII 9-10-EDA and FNIII 9-10-11-EDA were expressed, thus comprising the cell-binding domain with the synergy site^1^. To explore C-terminal extensions, FNIII 9-10-EDA-12-13-14 and FNIII 9-10-11-EDA-12-13-14 were expressed, thus comprising the FNIII 12-13-14 domain, which binds heparin^1^ and growth factors^20^. All FN fragments were successfully produced, and endotoxin levels were verified as being under 0.2 EU/μg using a limulus amebocyte lysate assay. Purity of the FN fragments is shown by SDS-PAGE in Fig. S1a.

### The immunostimulatory activity of FNIII EDA can be modulated by addition or deletion of neighboring domains

To explore the dependence of TLR4 agonization upon the presence of various neighboring domains in the FN protein chain, we characterized TLR agonization using THP1-Blue cells (derived from the human monocytic THP1 cell line and modified to contain a reporter of agonization of several TLRs) and HEK-Blue TLR4 cells (derived from the human embryonic kidney 293 cell line and modified to contain a reporter of agonization of TLR4) as well as cytokine expression in dendritic cells (DCs). As expected, FNIII EDA agonized TLR4 in THP1-Blue cells in a dose-dependent manner (Fig. 1b). Interestingly, addition of FNIII 11 at the N-terminus, i.e. FNIII 11-EDA, agonized TLR4 up to 4-fold more potently (P < 0.001) in THP1-Blue cells and up to 5-fold more potently (P < 0.001) in HEK-Blue TLR4 cells, compared to FNIII EDA (Fig. 1b, c). To explore if this gain of activity derived from intrinsic activity within FNIII 11 or from an effect of N-terminal extension, which could for example influence the stability of the FNIII EDA domain, we produced the alternative construct FNIII 9-10-EDA, i.e. containing a neighboring N-terminal extension with FN's cell-binding domain and synergy site yet not the N-terminal FNIII 11 domain. Surprisingly, FNIII 9-10-EDA agonized TLR4 to the same extent as FNIII 11-EDA in both cell lines. Moreover, the complete N-terminal construct, FNIII 9-10-11-EDA, showed statistically equivalent behavior (Fig. 1b, c). We further explored the impact of neighboring FNIII repeats by extending FNIII EDA to the C-terminus with the native structure FNIII 9-10-11-EDA-12-13-14, as well as FNIII 9-10-EDA-12-13-14. Unexpectedly, in both THP1-Blue and HEK-Blue TLR4 cells, C-terminal extension with the FNIII 12-13-14 domain abrogated TLR4 agonization (Fig. 1b, c). We then tested the ability of the different FNIII EDA-containing constructs to stimulate the production of inflammatory cytokines in DCs, which are key monitors of innate immune signals such as TLR4 agonists. DC expression of TNF-α and IL-12p70 closely paralleled the TLR4 agonization that was observed in THP1-Blue and HEK-Blue TLR4 cells. The N-terminal extension of FNIII EDA strongly enhanced cytokine response, while C-terminal extension with FNIII 12-13-14 strongly decreased it (Fig. 1d, e).

### The immunostimulatory activity of FNIII EDA is cryptic and dependent upon elastase 2 cleavage

We sought to understand the biological significance of the profound diminution of FNIII EDA activity through C-terminal extension with the FNIII 12-13-14 domain, hypothesizing that the FNIII EDA repeat is cryptic in its TLR4 agonizing activity. Using bioinformatics analytical tools^32^, we suspected the presence of an elastase 2 (a serine protease, also referred to as neutrophil elastase) substrate between FNIII EDA and FNIII 12, after ile_87_ in the 94 amino acid-long FNIII EDA domain. We verified the presence of such a cleavage site by incubating FNIII 9-10-EDA-12-14 with elastase 2. The digestion products were analyzed using Western blotting, detecting FNIII EDA or the 6xHis tag that is located at the C-terminus of the FN fragment. Therefore, the expected digestion products of the 67 kDa FNIII 9-10-EDA-12-14 protein are a 33 kDa FNIII EDA-positive fragment and a 34 kDa 6xHis tag-positive fragment. Western blotting confirmed the presence of the cleavage site, since both digestion products were detected (Fig. 1f).

To assess if the biological activity of FNIII 9-10-EDA-12-14 could be recovered after digestion by elastase 2, we characterized TLR4 agonization in the HEK-Blue TLR4 cells. Proteolytic enhancement of TLR4 agonization was observed (Fig. 1g), consistent with activity obtained with the recombinant fragments lacking FNIII12-14 although not as high. The elastase 2 enzyme itself did not activate TLR4 in this assay (Fig. 1g). Other proteases tested (MMP-2, MMP-3, MMP-9, thrombin and plasmin) did not show cleavage of the protein as indicated by SDS-PAGE analysis (Fig. S1b).

### Fibrin matrices functionalized with FNIII 9-10 + FNIII 11-EDA mediate CD8^+^ T cell expansion and effector phenotype

**Figure 2:**
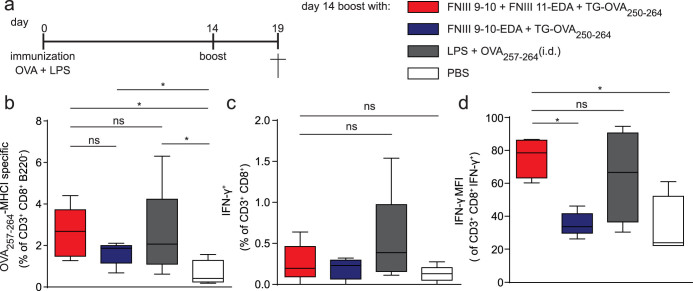
Fibrin matrices functionalized with FNIII 9-10 + FNIII 11-EDA stimulate an antigen-specific CD8^+^ T cell response. (a) Immunization schedule. C57BL/6J mice were immunized with 50 μg OVA and 20 μg LPS on day 0. The ability of FNIII EDA-containing fibrin-binding FN fragments to boost a CD8^+^ T cell response was tested on day 14 by implanting s.c. fibrin matrices functionalized with 5 nmol of TG-OVA_250–264_ (comprising the MHC-I binding immunodominant peptide of ovalbumin, OVA_257–264_) + 5 nmol of FNIII 11-EDA or FNIII 9-10-EDA (each with an N-terminal TG domain); alternatively mice were injected i.d. with 5 nmol of soluble OVA_257–264_ + 50 μg of LPS or with PBS only. On day 19, mice were sacrificed and the spleen was harvested. (b) Fibrin gels functionalized with fibrin-binding FNIII 9-10 + FNIII 11-EDA (each with an N-terminal TG domain) + TG-OVA_250–264_ induced levels of OVA_257–264_-MHC-I-specific CD8^+^ T cells in the spleen comparable to that induced by i.d. injections of LPS + OVA_257–264_. (c, d) IFN-γ production from splenocytes restimulated for 6 h *ex vivo* with OVA_257–264_ were evaluated by flow cytometry. The proportion of IFN-γ producing CD8^+^ T cell was similar among the different groups. However, the production of IFN-γ by CD8^+^ T cells was greater, as shown by the mean fluorescence intensity (MFI) of IFN-γ intracellular staining, in mice that were boosted with fibrin matrices functionalized with FNIII 9-10 + FNIII 11-EDA (each with an N-terminal TG domain) + TG-OVA_250–264_ or with LPS and OVA_257–264_; the group boosted with FNIII 11-EDA yielded statistically similar responses as the group boosted with LPS. Box plots represent median ± 95% confidence interval (n = 5). *P < 0.05; ns, not significant.

Since FNIII 11-EDA showed potent TLR4 activation *in vitro*, we evaluated its immunological activity *in vivo*. We selected a model based on the xenoantigen ovalbumin (OVA), in which vaccine-induced weak CD8^+^ T cell responses could potentially be further boosted (Fig. 2a). To evaluate boosting the CD8^+^ T cell responses, we co-delivered FNIII 11-EDA and the H2k^b^ MHC I immunodominant peptide OVA_257–264_^33^. Because FNIII EDA is naturally present within FN in the ECM, we decided to incorporate the FN fragments bound in matrix to mimic the *in vivo* situation. Moreover, this strategy allows creating an adjuvant-rich microenvironment, which has proved to be successful for cancer vaccination in other studies^34^. Here, as a matrix model, we used a fibrin hydrogel resembling a fibrin clot, with a volume of 150 μL and a concentration of 8 mg/mL of fibrinogen. To be incorporated in the matrix, the FN fragments were designed to contain a transglutaminase substrate sequence derived from α_2_-plasmin inhibitor (NQEQVSPL, denoted herein TG) at the N-terminus. Therefore, the FN fragments could be covalently incorporated in the matrix through the transglutaminase activity of factor XIIIa, which naturally crosslinks fibrin^35^. In addition, the OVA_257–264_ peptide was designed to be incorporated in fibrin. The TG sequence was fused to a longer sequence of OVA, namely OVA_250–264_, so as to comprise the protease substrate site SGLEQLE within the OVA chain that allows processing by APC antigen proteolysis machinery and liberation of the OVA_257–264_ epitope from TG-OVA_250–264_ for cross-presentation on MHC I^36^. Furthermore, because cell migration within fibrin is enhanced by integrin binding, we incorporated the major integrin-binding domain of FN, FNIII 9-10^37^. Two fibrin matrix compositions were tested: fibrin functionalized with FNIII 9-10 plus FNIII 11-EDA and fibrin functionalized with FNIII 9-10-EDA.

First, we gave an intradermal (i.d.) vaccine prime dose of soluble OVA adjuvanted with lipopolysaccaride (LPS), which resulted in strong CD4^+^ but modest CD8^+^ T cell responses, as indicated by OVA_257–264_:H2k^b^ p:MHC I pentamer binding (Fig. 2b). Boosting with the soluble MHC I epitope OVA_257–264_ + LPS increased the frequency of antigen-specific CD8^+^ T cells (P < 0.05). Boosting was also achieved by implanting fibrin matrices functionalized with FNIII 9-10 + FNIII 11-EDA + TG-OVA_250–264_ (P < 0.05) and with FNIII 9-10-EDA + TG-OVA_250–264_ (P < 0.05), although the boosting was marginally improved with the former (Fig. 2b). As an indication of the effector phenotype of these cells, we performed *ex vivo* restimulation of splenocytes with OVA_257–264_: although the frequency of IFN-γ-expressing CD8^+^ T cells was not enhanced by the FNIII EDA-containing proteins (Fig. 2c), the intensity of IFN-γ expression was enhanced by treatment with fibrin functionalized with FNIII 9-10 + FNIII 11-EDA (P < 0.05) (Fig. 2d).

### Fibrin matrices functionalized with FNIII 11-EDA ± FNIII 9-10 mediate functional cytotoxic T lymphocyte responses in the E.G7-OVA tumor model

To explore the extent to which FNIII 11-EDA-generated CD8^+^ T cells were capable of cell killing, i.e. function as cytotoxic T lymphocytes (CTLs), we employed a thymoma tumor model in which the cells express the xenoantigen OVA (E.G7-OVA). Tumor cells were injected subcutaneously (s.c.) in the back, and resulting tumors were allowed to reach a volume of 50 mm^3^. At that point, a fibrin matrix containing the FN fragments of interest and TG-OVA_250–264_ was implanted at a distant site at the level of the shoulder girdle close to the brachial lymph nodes or soluble OVA_257–264_ controls were injected i.d. in the four footpads, and delays in tumor growth were measured. Soluble controls were injected i.d. in the four footpads.

**Figure 3:**
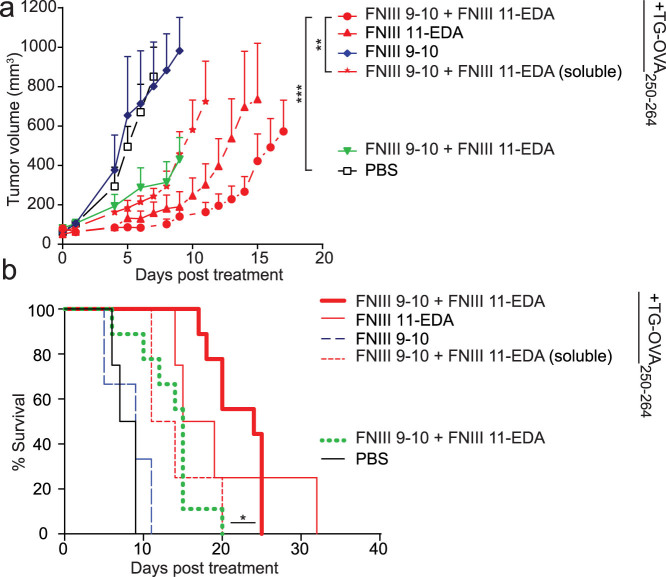
Single implantation of fibrin matrices functionalized with FNIII 9-10 + FNIII 11-EDA in E.G7-OVA tumor bearing mice reduces tumor growth rate. C57BL/6J mice were injected s.c. with 10^6^ E.G7-OVA cells on the back. When tumors reached 50 ± 5 mm^3^, the ability of fibrin-binding FNIII EDA-containing FN fragments to induce a functional CD8^+^ T cell response was tested by treating mice with fibrin matrices functionalized with various matrix formulations implanted s.c. or with soluble formulations injected i.d. Animals were sacrificed either when the tumor reached 1000 mm^3^ or for humane reasons. (a, b) Tumor growth was significantly delayed in animals vaccinated using a fibrin matrix functionalized with FNIII 11-EDA ± FNIII 9-10 (each with an N-terminal TG domain) + TG-OVA_250–264_ both in terms of tumor volume (a) and animal survival (b). TG-OVA_250–264_-free matrices also proved slightly effective in delaying tumor growth (a) and extending animal survival (b). Growth curves were stopped when the second animal of the corresponding group died, the value corresponding to the first animal which died was kept as a constant until the curve was stopped. Growth curves represent mean ± SEM; Kaplan-Meier survival-curves (n = 4–9). *P < 0.05; **P < 0.01; ***P < 0.001.

When the fibrin matrix was implanted only once, strong delays in tumor growth were observed with treatments comprising FNIII 11-EDA, especially in conjunction with FNIII 9-10 (Fig. 3a). Omission of FNIII 11-EDA from the fibrin matrix resulted in no delay of tumor growth. Although the TG-OVA_250–264_ peptide-free FNIII-EDA-containing matrix slightly reduced the tumor growth, the absence of antigen significantly reduced the response, demonstrating that the response is not mainly based on a general upregulation of immunity but is rather specific to the antigen co-incorporated in the fibrin matrix. Moreover, FNIII 9-10 + FNIII 11-EDA was not as effective when administered not bound to fibrin matrix, demonstrating functionality in the matrix-immobilized form. The Kaplan-Meier survival curves shown in Fig. 3b illustrate the benefits of co-delivery of FNIII 9-10 and FNIII 11-EDA with the tumor cell-specific peptide epitope. Although all tumors eventually reached 1000 mm^3^, the substantial delay in approaching this volume induced by a fibrin matrix with FNIII 11-EDA, especially in conjunction with FNIII 9-10, clearly indicates induction of functional CTL responses.

**Figure 4:**
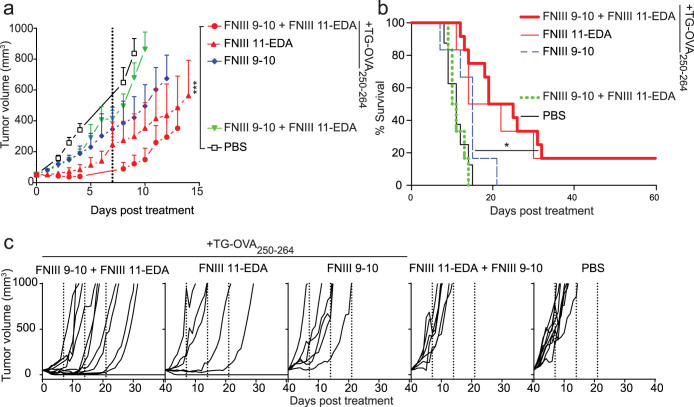
Multiple implantations of fibrin matrices functionalized with FNIII 11-EDA ± FNIII 9-10 reduces E.G7-OVA tumor growth rate and in cases mediates regression. C57BL/6J mice were injected s.c. with 10^6^ E.G7-OVA cells on the back. When tumors reached 50 ± 5 mm^3^, mice were treated weekly (day 0 and dashed lines) for three weeks with fibrin matrices functionalized with various formulations implanted s.c. Mice were sacrificed when the tumor reached 1000 mm^3^ or for humane reasons. Tumor growth was significantly delayed in animals treated using a fibrin matrix functionalized with FNIII 11-EDA ± FNIII 9-10 (each with an N-terminal TG domain) + TG-OVA_257–264_ both in terms of tumor volume (a) and animal survival (b). Individual growth curves (c) show regression in some of the animals treated with fibrin gels functionalized with TG-OVA_250–264_ and FNIII 11-EDA with or without FNIII 9-10 (each with an N-terminal TG domain). Growth curves were stopped when the second animal of the corresponding group died, the value corresponding to the first animal which died was kept as a constant until the curve was stopped. Growth curves represent mean ± SEM; Kaplan-Meier survival-curves (n = 6–12). *P < 0.05;***P < 0.001.

In the experiments described above, a single administration of a functionalized fibrin matrix was performed and the fibrin was slowly resorbed, whereas physiologically the FNIII EDA-containing FN proteolytic fragments would be present chronically. Thus, to further explore the ability of its chronic presence to induce CTL activity, we administered the functional fibrin matrices weekly after the tumors reached 50 mm^3^. In this model also, tumor growth was significantly delayed in animals treated with fibrin matrices functionalized with FNIII 11-EDA ± FNIII 9-10 + TG-OVA_250–264_ both in terms of tumor volume (Fig. 4a) and survival (Fig. 4b). Furthermore, none of the mice treated with PBS or with fibrin matrices lacking either FNIII 11-EDA or TG-OVA_250–264_ survived more than 21 days post treatment, whereas half of the mice treated with fibrin matrices functionalized with FNIII 11-EDA + TG-OVA_250–264_ ± FNIII 9-10 survived longer than 21 days and 20% even showed complete regression of the tumor, as shown in survival curves (Fig. 4b) and the individual growth curves (Fig. 4c). The efficacy of multiple injections of the soluble formulation was not assessed here as that approach already proved to be significantly less effective than functionalized fibrin matrix forms with a single administration. In this model of chronic exposure, co-treatment with FNIII 9-10 and FNIII 11-EDA yielded stronger CTL responses than mono-treatment with FNIII 11-EDA, both with the TG-OVA_250–264_ peptide antigen comprising the OVA_257–264_ epitope.

### Fibrin matrices functionalized with FNIII 9-10 + FNIII 11-EDA mediate functional cytotoxic T lymphocyte responses in the B16–F10 tumor model with an endogenous antigen

**Figure 5:**
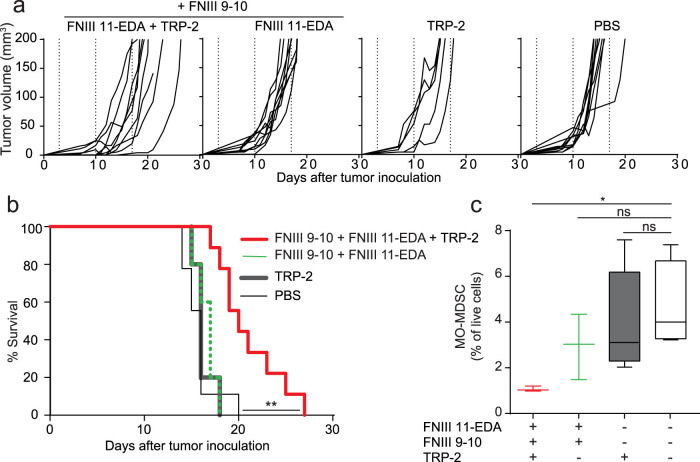
Multiple implantations of fibrin matrices functionalized with FNIII 9-10 + FNIII 11-EDA reduces B16-F10 melanoma tumor growth rate and modulates anti-tumor immune suppression. C57BL/6J mice were injected s.c. with 2.5 × 10^5^ B16–F10 cells on the back on day 0. On day 3, mice were treated weekly (dashed lines) for three weeks with fibrin gels functionalized with various formulations implanted s.c. Animal were sacrificed either when the tumor reached 200 mm^3^ or for humane reasons. Individual growth curves (a) and survival curves (b) show that tumor growth was significantly delayed in animals treated with fibrin matrices functionalized with FNIII 11-EDA + FNIII 9-10 (each with an N-terminal TG domain) + TG-TRP-2_173–188_, comprising the MHC-I immunodominant peptide epitope from the melanocyte-specific protein TRP-2 (TRP-2_180–188_). (c) The percentage of monocytic myeloid-derived suppressor cells (MO-MDSC, flow-cytometry gated as being CD11b^+^CD11c^-^MHC-II^-^Ly6C^hi^Ly6G^-^) in the spleen was significantly reduced in the mice treated with the fibrin gel containing and FNIII 9-10 + FNIII 11-EDA (each with an N-terminal TG domain) + TG-TRP-2_173–188_ compared to the naïve control. Graphs show Kaplan-Meier survival-curves (n = 6–12). Box plots represent median ± 95% confidence interval (n = 3–5). *P < 0.05; **P < 0.01.

In the experiments with E.G7-OVA cells, the surrogate tumor antigen OVA is a xenoantigen, not centrally tolerized in the mouse. To explore the activity of the FNIII 11-EDA domain to induce immunological responses versus a centrally tolerized antigen, we used the B16–F10 melanoma model. Fibrin matrices were functionalized with FNIII EDA-containing FN fragments and with the MHC I immunodominant epitope from tyrosinase-related protein-2 (TRP-2), namely TRP-2_180–188_^38^. As with the OVA-derived epitope, we designed the TRP-2-derived epitope to contain a longer peptide sequence from TRP-2 for proteolytic processing of the antigen and an N-terminal TG sequence for immobilization in fibrin, namely TG-TRP-2_173–188_. Mice were treated starting from the third day after tumor inoculation, when the tumors were palpable, and were further treated weekly for three weeks. Consistent with the results in the E.G7-OVA model, tumor growth was significantly delayed in animals treated with the fibrin matrix functionalized with FNIII 11-EDA + FNIII 9-10 + TG-TRP-2_173-188_, as shown by the individual (Fig. 5a) and aggregate (Fig. 5b) growth curves, as well as the Kaplan-Meier survival curves (Fig. 5c). As in the experiments with the E.G7-OVA cells, the combination of the antigen with either FNIII 11-EDA or FNIII 11-EDA + FNIII 9-10 yielded similar results; only the latter, which proved slightly more effective at reducing the initial tumor volume, was retained for testing against the aggressive B16–F10 model.

The B16–F10 melanoma model is known as an immune suppressive model, owing in part to the infiltration of monocytic myeloid-derived suppressor cells (MO-MDSCs) in the tumor as well as their proliferation in the spleen^39^,^40^. Therefore, we examined MO-MDSC numbers in the spleens of the B16-F10-bearing mice. Mice receiving the fibrin matrices containing TG-TRP-2_173–188_ but no FNIII fragments showed the same number of splenic MO-MDSCs compared to untreated mice or mice treated with fibrin matrices functionalized with FNIII 11-EDA + FNIII 9-10 without TG-TRP-2_173–188_. However, mice treated with fibrin matrices functionalized with FNIII 11-EDA + FNIII 9-10 + TG-TRP-2_173–188_ showed a substantial reduction in the number of splenic MO-MDSCs, demonstrating the ability of the FNIII 11-EDA domain to induce local (delay of tumor growth) and systemic (reduction in splenic MO-MDSC numbers) immune responses to an endogenous antigen, here the melanocyte-specific antigen TRP-2.

## Discussion

Although roles of FN in processes such as embryogenesis and wound healing have been described^41^,^42^, FN's interaction with the immune system is not well understood. The FNIII EDA domain, present in a splice variant of FN, which is found in sites of transient inflammation as in tissue damage^4^,^5^,^6^ and of chronic inflammation such as psoriatic lesions^10^,^11^,^12^ and scleroderma lesions^15^, has been shown to agonize TLR4^14^. In the case of scleroderma, for example, agonization of TLR4 was shown to drive a cycle of fibrosis, leading to further FNIII EDA-containing FN expression, increasing TLR4 agonization and promoting continued fibrosis^15^; as such, TLR4 agonization via FNIII EDA is central to the pathophysiology of the disease. Moreover, because of its ability to activate TLR4, FNIII EDA has been explored in cancer vaccinology, utilizing FNIII EDA-antigen fusion proteins as DC-targeting adjuvants to induce anti-tumor CTLs^28^,^30^,^31^. In this work, at least part of the effect of the FNIII EDA domain has been attributed to targeting the antigen for DC uptake through binding to TLR4^28^,^30^. Nevertheless, FNIII EDA showed anti-tumor potency only in combination with other TLR ligands.

In our study, we first sought to explore the dependence of FNIII EDA's agonization of TLR4 on its intramolecular context, addressing roles played by the N-terminally neighboring FNIII 11 domain and FNIII 9-10 domain, which binds cell-surface integrins α_5_β_1_ and α_v_β_3_^1^,^16^,^17^,^43^, and by the C-terminally neighboring domain FNIII 12-13-14, which binds heparin and heparan sulfate^19^ and growth factors^21^. Moreover, we hypothesized that the agonization of TLR4 by FNIII EDA could be cryptic, because some of the physiological mechanisms involving FN have been shown to be carried out by cryptic sites^44^,^45^,^46^,^47^ requiring conformational modification or proteolytic cleavage to exert their functions, including stimulation of MMP expression by FNIII EDA^24^. Thus, we were also motivated to search for proteases that might enhance FNIII EDA activity.

We expressed a family of FN fragments (Fig. 1a) comprising the FNIII EDA repeat with various N-terminal and C-terminal extensions, so as to include FNIII 9-10, FNIII 11, and FNIII 12-13-14. Then, we characterized the ability of these recombinant FN fragments to agonize TLR4, activate DCs, stimulate CD8^+^ T cell expansion, and induce functional CTLs, where functionality was judged by cytotoxicity in a tumor vaccine model. Our *in vitro* results show that extension of the FNIII EDA domain to the N-terminus enhances its ability to agonize TLR4 (Fig. 1b, c) and induce inflammatory cytokine expression from DCs (Fig. 1d, e). This phenomenon was not sensitive to the details of the FNIII domain to the N-terminus, as extensions with FNIII 11, FNIII 9-10, and FNIII 9-10-11 yielded equivalent results (Fig. 1b, e). Thus, we conclude that co-binding of integrins α_5_β_1_ and α_v_β_3_ is likely not involved in the effect. Rather, the observed 4–5 fold enhancement of TLR4-agonization activity may be due to structural stabilization of the FNIII EDA repeat, which, like other FNIII domains, is not stabilized by disulfide bonding. It is noteworthy that agonization of TLR4 by N-terminally extended FNIII EDA is very potent, comparable to that achieved by the prototypical microbial TLR4 agonist LPS (Fig. 1b), yet without the cytotoxicity that is accompanied by high doses of LPS, which leads to lower NF-κB signaling^48^ (Fig. 1b).

The possibility that our fragments could be contaminated with LPS was excluded by conducting these experiments in the presence of polymyxin B, which binds and inactivates LPS^49^,^50^; moreover, the expression and purification protocol for FNIII EDA (observed to be a poor agonist) and FNIII 11-EDA, FNIII 9-10-EDA, and FNIII 9-10-11 EDA (good agonists) were very similar. Finally, LPS endotoxin levels were tested in all the recombinant FN fragments and were found to be below 0.2 EU/μg in each.

Considering the C-terminal context of FNIII EDA, the existence of a protease substrate was suggested between the FNIII EDA and FNIII 12 domains using a bioinformatic analysis^32^, specifically at the C-terminal end of FNIII EDA (position 87 of 94 total). Experimentally, elastase 2 (neutrophil elastase) was observed to cleave between FNIII EDA and FNIII 12 (Fig. 1f), and cleavage was shown to enhance TLR4 agonization compared to the intact control (Fig. 1g). Returning to the observation of the role of FNIII EDA and TLR4 in lesions such as in psoriasis^10^,^11^,^12^ and scleroderma^15^, which are characterized by neutrophil infiltration^51^,^52^, it may be that neutrophil-derived elastase-2-mediated proteolysis of FN to potentiate a cryptic TLR4-agonizing site is involved in the pathophysiological mechanism.

Although FN is present in the serum, its main biological activities are carried out in immobilized form as a component of the ECM. Thus, we also sought to investigate FNIII EDA's immunological activity when immobilized in a matrix. As previously shown by our laboratory, recombinant proteins bearing an enzymatic substrate for the coagulation transglutaminase Factor XIIIa can be covalently incorporated into fibrin matrices^53^, a method that has proven to be an efficient delivery method for integrin ligands^37^ and growth factors in the context of tissue repair^20^,^22^,^37^. To determine whether FNIII EDA would induce similar reactions while presented in a more natural environment, we produced ECM-mimicking fibrin matrices functionalized with FNIII EDA-containing FN fragments and with the MHC I immunodominant peptide from OVA as a model antigen. In a first experiment aiming to test antigen-specific CD8^+^ T cell expansion, exposure to fibrin matrices functionalized with FNIII 11-EDA and TG-OVA_250–264_ induced expansion (Fig. 2b) with more intense IFN-γ production upon re-stimulation with antigen (Fig. 2d). Interestingly, the effect of the engineered matrix was comparable to that achieved by administration of OVA_257–264_ (which does not require antigen processing) adjuvanted with LPS (Fig. 2d).

Surprisingly, fibrin matrices functionalized with FNIII 9-10-EDA induced significantly lower responses than matrices functionalized with both FNIII 11-EDA and FNIII 9-10. Such a difference may be due to FNIII 9-10-EDA's tendency to precipitate at the concentrations used for the preparation of the fibrin matrices. These results show that FNIII EDA-rich ECM analogs can enhance a pre-established immune response at the level of antigen-specific CD8^+^ T cell expansion.

Because FNIII EDA has been shown to induce cellular immune responses including activation of CD8^+^ T cell responses, the domain in soluble form^26^,^27^,^28^,^29^,^30^,^31^ has been explored extensively as a cancer vaccine adjuvant in mouse models including when antigens are fused to it^28^,^30^,^31^, although most potently in combination with other TLR agonists^28^,^31^. Notably, when FNIII EDA-antigen fusions have been used as cancer vaccines, in conjugation with other TLR agonists, FNIII EDA has been used without N-terminal extension and as a soluble protein (i.e. not linked to the ECM)^28^,^30^,^31^. Here, we employed tumor cell-killing assays to characterize the functionality of ECM-bound FNIII EDA's effect upon CD8^+^ T cells to act as CTLs. Two models were utilized, one with a xenogeneic model tumor antigen, OVA (the E.G7-OVA thymoma model; Fig. 3 and 4), where the antigen is not centrally tolerized, and one with an endogenous tumor antigen TRP-2 (B16-F10 melanoma; Fig. 5), in which central tolerance must be overcome. The B16F10 melanoma model progresses very quickly, and as such the rate of tumor growth may outpace the rate of the adaptive immune response. In both models, administration of matrix-bound antigen (TG-OVA_250–264_ or TG-TRP-2_173–188_) with matrix-bound FNIII 11-EDA induced substantial delays in tumor growth, without any additional TLR agonists. Furthermore, while the addition of FNIII 9-10 did not improve the survival time of the mice, it nonetheless helped reduce the average tumor volume. Moreover, in the case of the E.G7-OVA model with repeated matrix implantation, complete tumor remission was observed in some instances (Fig. 4), however it is not known if the eventual escape in other animals from immune killing occurs via a *bona fide* immunological mechanism or due to selective pressure of vaccination toward antigenic selection leading to elimination of the OVA-encoding plasmid^54^,^55^.

To demonstrate that a prolonged presence of the FNIII EDA domain bound to the fibrin matrix was beneficial, the same molecules in soluble form were injected i.d (Fig. 3). This control route was chosen, because it allows for an efficient delivery of the vaccine to the same skin-draining lymph node as the tumor (in addition to other non-affected lymph nodes) and thus maximizes the effect via a high percentage of targeted APCs being in contact with both the tumor antigens and the vaccine^56^. However, i.d. injection was much less effective in delaying tumor growth (Fig. 3) compared to the fibrin matrix, clearly demonstrating that matrix binding of FNIII EDA fragment is beneficial. Furthermore, we judged the role of the inflammation from the surgical implantation of the matrix as being negligible, as FNIII-11-EDA-free matrices failed to provide protection against the tumor despite the presence of TG-OVA_250–264_ (Fig. 3). Thus, using two tumor models, one with an exogenous model antigen and one with an endogenous antigen, N-terminally-extended FNIII EDA without C-terminal extension with the native FNIII 12-13-14 domain was shown to induce potent immunity as characterized by the cytotoxic functionality of induced CTLs when the FN domains were co-administered in a matrix with antigen. Moreover, the ability to kill the syngeneic melanoma demonstrates the ability to induce functional autoimmunity.

Furthermore, in addition to exploring the ability of FNIII EDA to modulate immunity at the site of its presence, we explored effects in the spleen. The B16–F10 model is known to be immune suppressive through induction of MO-MDSCs, which traffic between the tumor and the spleen^57^. Administration of fibrin matrixes presenting FNIII 11-EDA, FNIII 9-10 and TG-TRP2_173–188_ resulted in a substantial diminution of the frequency of MO-MDSCs in the spleen, demonstrating an ability to alter this immune suppressive mechanism (Fig. 5c).

In conclusion, these results bring insight into the immunological function of the FNIII EDA-containing FN splice variant *in vivo*. We revealed that the TLR4 agonizing potential of FNIII EDA is cryptic in FN, becoming exposed by cleavage between FNIII EDA and FNIII 12 by the neutrophil elastase 2. Lacking the C-terminal FNIII 12-13-14 domain, TLR4 agonization was potentiated up to 5 fold. Moreover, by using two tumor growth models, we demonstrated that induction of CTL responses to a xeno- and an auto-antigen by FNIII EDA activity is enhanced when the domain is bound to the an ECM analog. Therefore, because the FNIII EDA-containing splice variant is expressed in sites of chronic inflammation, autoimmunity, and in many tumors^10^,^11^,^12^, this ECM protein may contribute to the pathology and as well to an anti-tumoral benefit to the immune microenvironment by agonizing TLR4 especially after elastase-2-mediated proteolysis. In addition, delivering ECM-bound FNIII EDA fragments in combination with antigens could be an attractive option for anti-tumoral immunotherapies.

## Methods

### Recombinant proteins

All FN fragments were engineered to bear at their N-terminus the coagulation transglutaminase Factor XIIIa peptide substrate NQEQVSPL (TG) and were cloned in a pET-22b (+) (Novagen) plasmid between the restriction sites NdeI and NotI. This plasmid contains a 6xHis-tag being added at the 3′ end of the inserted genes. *E. coli* BL21DE3 were transformed and used for protein production. The recombinant proteins were purified using a HIStrap column (GE Healthcare) and washed using 0.1% Triton X-100 in PBS for LPS removal and an ATP solution (2 mM ATP, 50 mM Tris-HCl, 10 mM MgSO_4_, pH 7.4) for DnaK removal. An imidazole (1 M) buffer was used for elution. The proteins were then stored in TBS at −80°C. Endotoxin levels were tested by Quantitative Chromogenic Limulus Amebocyte Lysate and were below 0.2 EU/μg.

### Reagents

Ultrapure LPS Escherichia coli 0111:B4 was purchased from InvivoGen. Low endotoxin grade OVA (<0.01 EU/μg protein) was used for immunization (Hyglos). MHC-I H-2Kb immunodominant peptides OVA_257–264_ (SIINFEKL) and TRP-2_180–188_ (SVYDFFVWL) were ordered from GenScript with the TG-sequence at their N-termini followed with N-terminal extension from the native protein OVA (SGLEQLE)^36^ allowing proteolytic processing to liberate the epitopes (NQEQVSPLSGLEQLESIINFEKL, NQEQVSPLSGLEQLESVYDFFVWL). PE-labeled H-2Kb/OVA_257–264_ pentamer was purchased from ProImmune. Pentamer staining was performed according to the manufacturer's instructions.

### Western blotting

FNIII 9-10-EDA-12-13-14 was first incubated with elastase 2 (Merck) at a 50:1 ratio for 3 h at 37°C in PBS. SDS-PAGE (12% polyacrylamide gels) was then performed, followed by transfer to a nitrocellulose membrane. The membrane was then incubated either with an HRP-conjugated anti-6xHis (abcam) or with an anti-FNIII EDA (abcam, IST-9) antibody followed by an HRP-conjugated goat anti-mouse IgG1 secondary antibody (SouthernBiotech). After incubation, the blot was processed with SuperSignal West Pico Chemiluminescent Substrate (Pierce).

### *In vitro* stimulation of bone marrow-derived dendritic cells

Murine bone marrow-derived DCs were generated as described elsewhere^58^. On day 8, cells were plated at a density of 5 × 10^5^ cells/well in 96-well plates and incubated overnight with 0.5 μM of recombinant proteins pre-incubated with 10 μg/ml of polymixin B to block interactions with any potentially contaminating LPS. Cytokines released in the supernatant were measured by ELISA (eBioscience).

### *In vitro* stimulation of HEK-Blue TLR4 cells

HEK-Blue TLR4 LPS Detection Kit (Invivogen) containing HEK293 cells are engineered to express TLR4, MD2 and CD14 as well as a secreted embryonic alkaline phosphatase (SEAP) reporter gene controlled by an NF-κB-inducible promoter. HEK-Blue TLR4 cells were plated at a density of 10^4^ cells/well in 48-well plates and were cultured for 48 h until they were 80% confluent. They were then incubated overnight with 0.5 μM of recombinant proteins pre-incubated with 10 μg/ml of polymixin B. All protein solutions and an elastase 2 control were incubated 3 h at 37°C before stimulation. FNIII 9-10-EDA-12-13-14 + elastase 2 was prepared in a 50:1 ratio. After incubation, the supernatant was diluted 1:10 in HEKBlue Detection Medium (Invivogen) and the expression of SEAP was measured by light absorption at 620 nm. Cell culture medium was DMEM + 10% FBS and assay medium was DMEM + 2% FBS.

### *In vitro* stimulation of THP1-Blue cells

THP1-Blue cells (Invivogen) are human THP-1 monocyte cell line engineered to express an NF-κB-inducible SEAP reporter construct. THP1-Blue cells were plated at a density of 5 × 10^5^ cells/well in 96-well plates and incubated overnight with two-fold serial dilutions of recombinant proteins pre-incubated with 10 μg/ml of polymixin B or LPS ranging from 0.012 μM to 12.5 μM and 0.06 ng/μl to 30 ng/μl, respectively. After incubation, the supernatant was diluted 1:10 in HEKBlue Detection Medium (Invivogen) and the expression of SEAP was measured by light absorption at 620 nm. Cell culture medium was RPMI 1640 (2 mM L-glutamine, 1.5 g/L sodium bicarbonate, 4.5 g/L glucose, 10 mM HEPES and 1.0 mM sodium pyruvate) with 10% heat inactivated FBS supplemented with 100 μg/ml of Zeocin and assay medium was RPMI 1640 + 2% FBS.

### Mice and tumor cells

C57BL/6J mice (aged 7–8 wk) were purchased from Harlan Laboratories and kept under pathogen-free conditions at the animal facility of Ecole Polytechnique Fédérale de Lausanne. All experiments were performed in accordance with Swiss law and with approval from the Cantonal Veterinary Office of Canton de Vaud, Switzerland. E.G7-OVA thymoma cells (i.e., EL4 cells (a thymoma line) expressing OVA as a surrogate tumor antigen, ATCC TIB-39) were obtained from American Type Culture Collection (ATCC) and grown in RPMI medium 1640 (Invitrogen) supplemented with 10% heat-inactivated FBS, 1 mM sodium pyruvate, 30 mM Hepes, 50 μM 2-mercaptoethanol, and 0.4 mg/mL G418 antibiotic (Sigma). B16–F10 (ATCC CRL-2539) melanoma cells were obtained from American Type Culture Collection (ATCC) and were maintained in DMEM supplemented with 10% (vol/vol) FBS and penicillin/streptomycin/amphotericin B.

### Preparation of functionalized fibrin matrices

Fibrin matrices (150 μL) were formed by mixing a fibrinogen solution with a thrombin solution of equal volume in 1.5 mL Eppendorf tubes. The fibrin solution contained 16 mg/mL fibrinogen (plasminogen-, von Willebrand factor- and FN-depleted; Enzyme Research Laboratories) in HEPES buffer (20 mM HEPES, 150 mM NaCl, pH 7.4). The thrombin solution contained, 2 U/mL human thrombin (Sigma–Aldrich), 5 nmol of TG-OVA_250–264_ and/or 5 nmol of FNIII 11-EDA or FNIII 9-10-EDA, and 5 mM Ca^2+^ in HEPES buffer.

### Stimulation of antigen-specific CD8^+^ response

C57BL/6J mice were immunized by injection in the four footpads with a total dose of 50 μg OVA and 20 μg LPS on day 0. On day 14, fibrin gels functionalized with 5 nmol of TG-OVA_250–264_ and 5 nmol of FNIII 11-EDA or FNIII 9-10-EDA were implanted s.c. on the back at the level of the shoulder girdle; alternatively mice were injected i.d. with 5 nmol of OVA_257–264_ and 50 μg of LPS or plain PBS. On day 19 mice were sacrificed and the spleen was harvested.

### Single-cell preparation and *ex vivo* antigen-specific cell restimulation

Splenocytes were obtained by squeezing the spleen with a razor blade and disrupting the cell aggregates on a cell sieve, washing thoroughly, and lysing the red blood cells. For CD8^+^ T cell antigen-specific restimulation and intracellular cytokine staining, immune cells were cultured at 37°C for 6 h in the presence of 1 μg/mL OVA_257–264_ peptide. All cells were cultured in IMDM medium supplemented with 10% FBS and 1% penicillin/streptomycin (all from Invitrogen).

### Flow cytometry and ELISA

Before antibody staining for flow cytometry, all cells were labeled with live/dead fixable cell viability reagent in PBS (Invitrogen). For surface staining, cells were incubated for 15 min with the antibodies diluted in HBSS (Invitrogen)/0.5% BSA (PAA Laboratories). For antigen specificity, cells were stained with a SIINFEKL:H2Kb MHC I pentamer (ProImmune). For intracellular cytokine staining, cells were fixed in 2% paraformaldehyde solution, washed with 0.5% saponin (Sigma-Aldrich) in HBSS/0.5% BSA solution, and incubated with the antibodies diluted in saponin solution for 30 min. After washing, cells were resuspended in HBSS/0.5% BSA solution for analysis. Samples were acquired on CyAn ADP Analyzer (Beckman Coulter) and data were analyzed with FlowJo software (Tree Star). The antibodies anti-mouse CD11c, CD62L, B220, CD44 and IFN-γ were purchased from BioLegend; anti-mouse CD3_ε_, CD8, CD11b, Ly6c, Ly6g, TNF-α and MHC-II antibodies were purchased from eBioscience (San Diego, CA, USA). Ready-SET-go! ELISA kits for cytokine detection were purchased from eBioscience and used according to manufacturer's instructions.

### Tumor growth assays

Tumor cells were implanted s.c. in the back at the level of the junction between the thoracic and lumbar vertebrae of C57BL/6J mice with inoculation of 10^6^ E.G7-OVA cells or 2.5 × 10^5^ B16–F10 cells in 30 μL PBS (Invitrogen). The mice were then treated once (E.G7-OVA) or weekly for three weeks (E.G7-OVA and B16–F10) when their tumor reached 50 mm^3^ (E.G7-OVA) or when the tumor became visible (B16–F10). Fibrin gels (150 μl total, fibrinogen (8 mg/ml)) were functionalized with 5 nmol of FNIII domain recombinant proteins and with or without 5 nmol of MHC I immunodominant peptide bound to the fibrin, as described above. Tumors were measured 5 times per week and volumes were calculated as ellipsoids based on three orthogonal measures. Animal were killed either when the tumor reached 200 mm^3^ (B16–F10) or 1000 mm^3^ (E.G7-OVA) or for humane reasons such as tumor necrosis, excessive loss of weight or evident isolation from the other animals.

### Statistical Analyses

Statistical analyses were performed using Analysis of Variance (ANOVA) and Bonferonni posttest using GraphPad Prism.

## Author Contributions

Z.J., M.M.M., L.J. and J.A.H. designed research; Z.J. and A.d.T. performed research; Z.J., M.M.M. and A.d.T. analyzed data; J.A.H. directed the project; and Z.J., M.M.M. and J.A.H. wrote the paper.

## Supplementary Material

Supplementary Information Figure S1
