# Supplementary material for: The TLR4 Agonist Fibronectin Extra Domain A is Cryptic, Exposed by Elastase-2; use in a fibrin matrix cancer vaccine
Source: Sci Rep. 2015 Feb 24;5:8569. doi: 10.1038/srep08569 (PMC4338432; doi:10.1038/srep08569)
Supplement: Supplementary Information — Figure S1 [file srep08569-s1.pdf]

The TLR4 Agonist Fibronectin Extra Domain A is Cryptic, Exposed by Elastase-2; use in a fibrin matrix cancer vaccine

Ziad Julier[a,1], Mikaël M. Martino[a,b,1], Alexandre de Titta[a], Laura Jeanbart[a], Jeffrey A. Hubbell[a,c,d,e,*]

Author affiliations: [a] Institute of Bioengineering, Ecole Polytechnique Fédérale de Lausanne, 1015 Lausanne, Switzerland; [b] World Premier International Immunology Frontier Research Center, Osaka University, Suita, Osaka 565-0871, Japan; [c] Institute for Chemical Sciences and Engineering, Ecole Polytechnique Fédérale de Lausanne, 1015 Lausanne, Switzerland; [d] Institute for Molecular Engineering, University of Chicago, Chicago, IL 60637, USA; [e] Materials Science Division, Argonne National Laboratory, Argonne, IL 60439, USA

[1] These authors contributed equally

[*] To whom correspondence should be addressed, jeffrey.hubbell@epfl.ch or jhubbell@uchicago.edu

Supporting Information

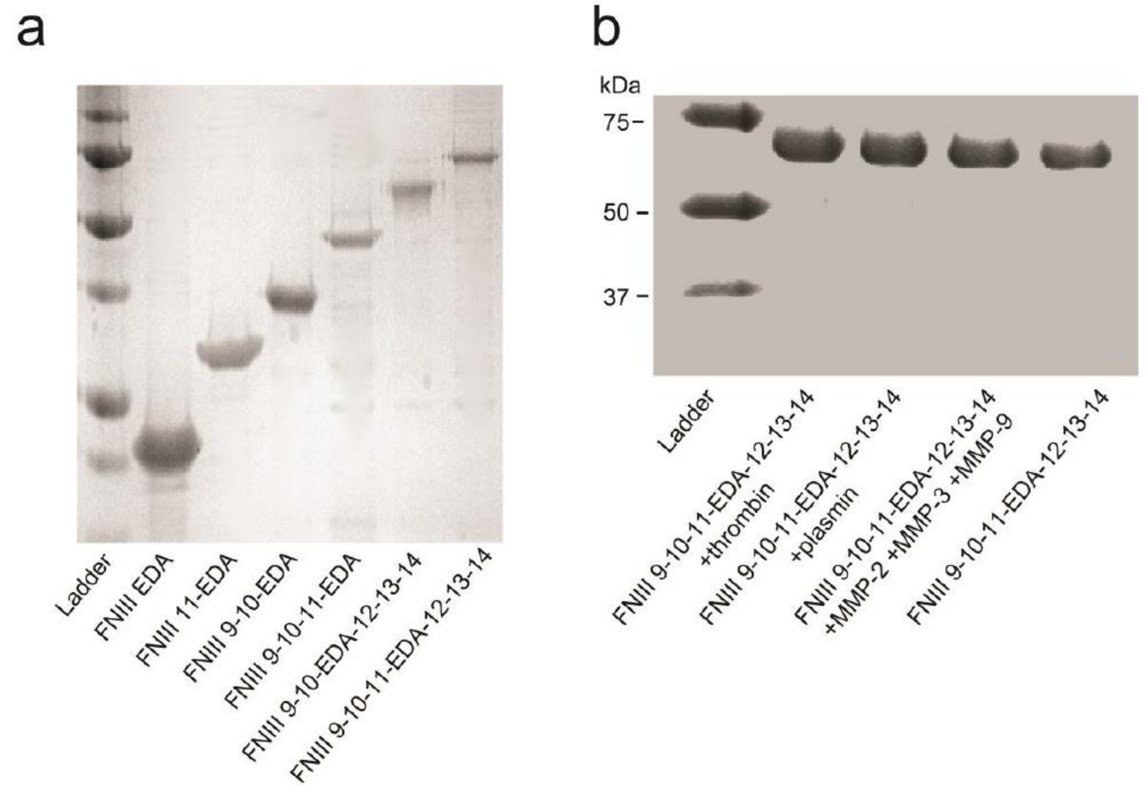

Fig. S1 (a) SDS-PAGE analysis of the 6 different FNIII EDA-containing FN fragments produced. (b) SDS-PAGE of FNIII 9-10-EDA-12-13-14 with or without prior incubation with thrombin or plasmin or a cocktail of MMP-2, MMP-3 and MMP-9
